# Supplementary material for: Cdk1-mediated DIAPH1 phosphorylation maintains metaphase cortical tension and inactivates the spindle assembly checkpoint at anaphase
Source: Nat Commun. 2019 Feb 28;10:981. doi: 10.1038/s41467-019-08957-w (PMC6395754; doi:10.1038/s41467-019-08957-w)
Supplement: Supplementary file 1 — Supplementary Information [file 41467_2019_8957_MOESM1_ESM.pdf]

SUPPLEMENTARY INFORMATION

Cdk1-mediated DIAPH1 phosphorylation maintains cortical tension during metaphase, which regulates inactivation of the spindle assembly checkpoint at anaphase onset

Nishimura et al.

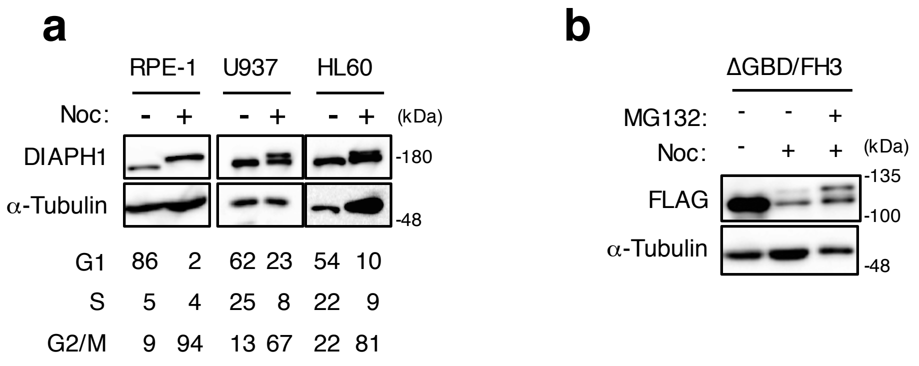

Supplementary Figure 1. Endogenous DIAPH1 is phosphorylated in multiple cell lines

(a) RPE-1, U937 and HL60 cells were collected in the presence (+) or absence (-) of 100 ng/ml nocodazole for 18 hrs, followed by mitotic shake-off (RPE-1). Cell lysates were subjected to immunoblotting. Cell cycle synchronization was monitored by FACS analysis. **(b)** HeLa cells transduced with 3×FLAG-DIAPH1-ΔGBD/FH3 mutant were synchronized by nocodazole and mitotic shake-off as described in a, in the presence (+) or absence (-) of 20 mM MG-132 treatment for 8 hrs. Cell lysates were subjected to immunoblotting.

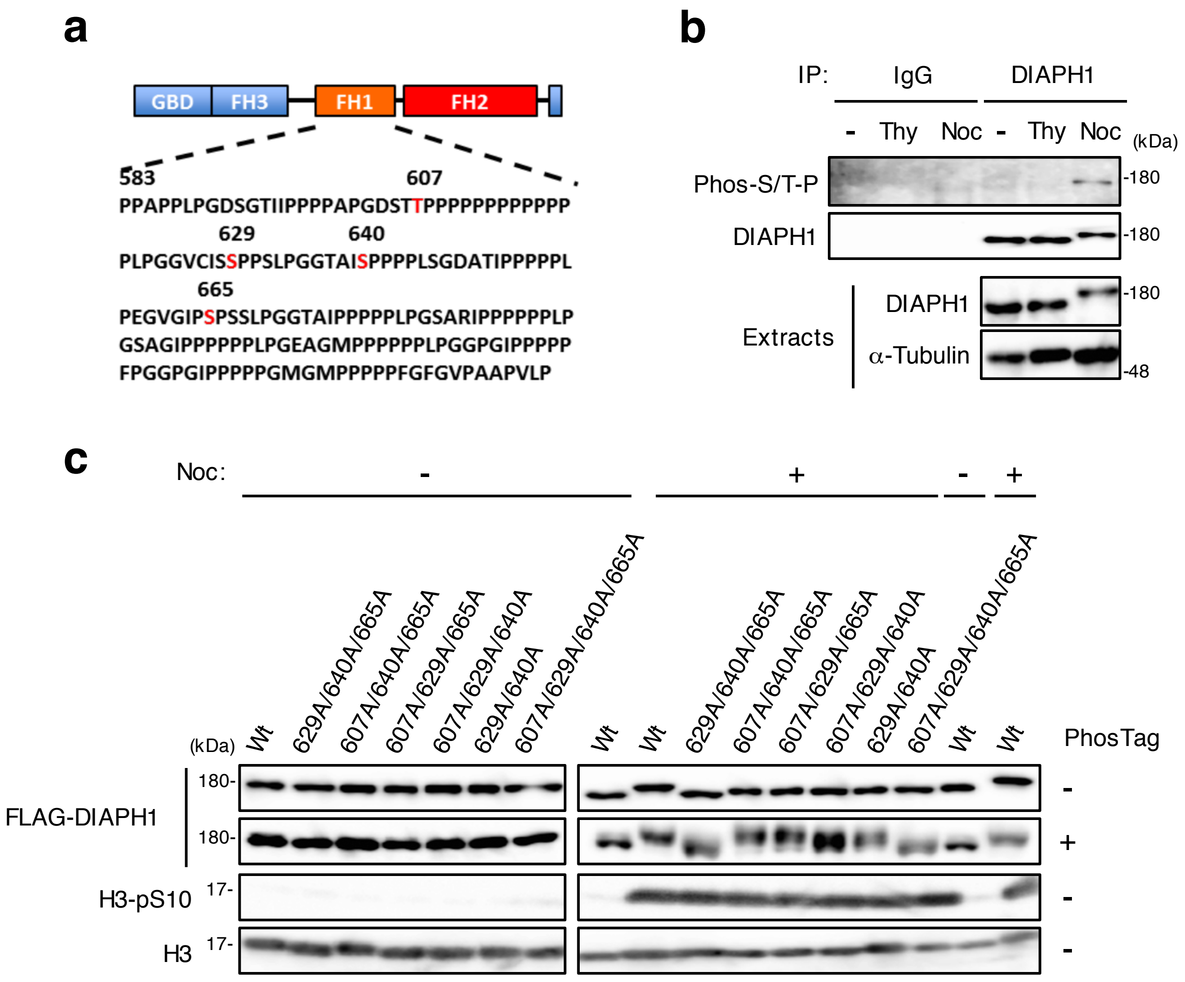

Supplementary Figure 2. S/T-P sites within the FH1 domain are phosphorylated during mitosis

(a) Amino acid sequence of the FH1 domain. S/T-P sites are shown in red. **(b)** HeLa cells were synchronized by double thymidine treatment (2.5 mM) for 16 hrs plus 8 hrs (Thy) or nocodazole treatment (100 ng/ml) for 18 hrs, followed by mitotic shake-off (Noc). Cell lysates were subjected to immunoprecipitation with mouse IgG or anti-mDia1 and immunoblotting. (-), asynchronous culture. **(c)** HeLa cells expressing 3×FLAG-DIAPH1-Wt or DIAPH1 mutants were synchronized by nocodazole treatment and mitotic shake-off as in **(a)**. Cell lysates were subjected to Phos-tag SDS-PAGE and immunoblotting using the indicated antibodies.

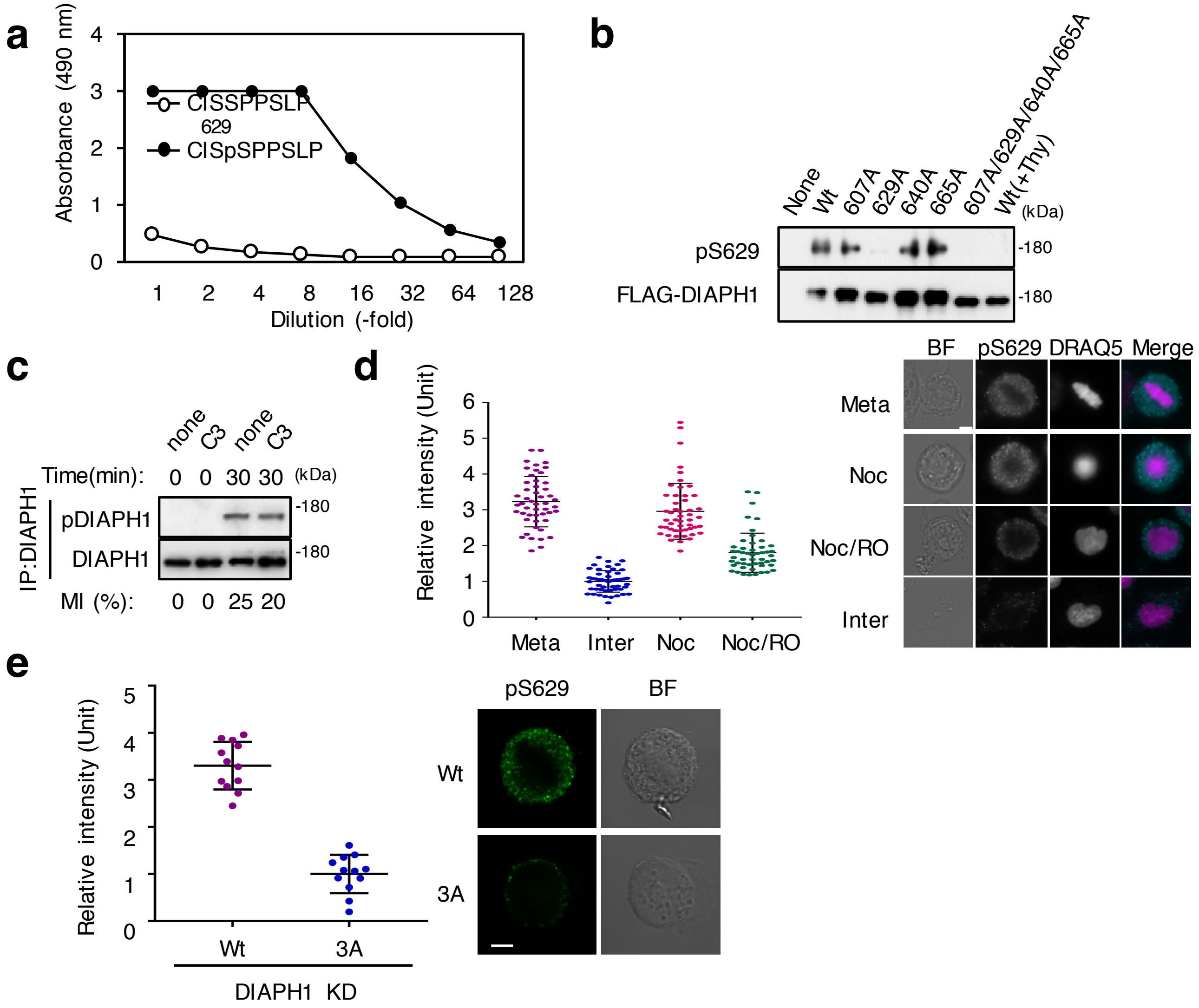

Supplementary Figure 3. Phospho-specific antibodies recognize p-DIAPH1 at S629

(a) Specificity of antibodies to phosphorylated DIAPH1 at S629 was evaluated by ELISA using phospho- or unphospho-peptides as indicated. **(b)** Hela cells expressing 3×FLAG-DIAPH1-Wt or its mutants were synchronized by nocodazole, followed by mitotic shake-off or with 2.5 mM thymidine (+Thy). Cell lysates were then subjected to immunoprecipitation using anti-FLAG antibody followed by immunoblotting using anti-phospho-Ser629 antibodies. **(c)** HeLa cells were synchronized at G2 phase and then released into fresh medium with or without 2 μg/ml C3 transferase. Samples were collected at the indicated times, and subjected to immunoprecipitation with anti-DIAPH1 followed by immunoblotting with the indicated antibodies. Mitotic cells were monitored by DAPI staining and mitotic index (MI%) was determined as percentages of total cell (n=100). **(d)** Asynchronous HeLa cells (Meta, Inter) or HeLa cells treated with nocodazole (Noc) or nocodazole followed by RO-3306 treatment (Noc/RO) were fixed with formaldehyde and immunostained using anti-pS629 antibodies. Chromosomes were visualized by DRAQ5 staining. Left: relative signal intensity (mean) of the cytosolic signal was quantified. Black bars indicate means ±SD. Right: representative images are shown. Scale bar, 5 μm. n=50 for each phase. **(e)** Asynchronous DIAPH1-knockdown HeLa cells (DIAPH-KD) expressing DIAPH1-Wt or -3A were fixed with formaldehyde and then subjected to immunostaining using anti-pS629 antibodies. Chromosomes were visualized by DAPI staining. The cells undergoing mitotic cell rounding were analyzed. Left: relative signal intensity (mean) of the cytosolic signal was quantified. Black bars indicate means ±SD. Right: representative images are shown. Scale bar, 5 μm. Wt: n=12, 3A: n=12.

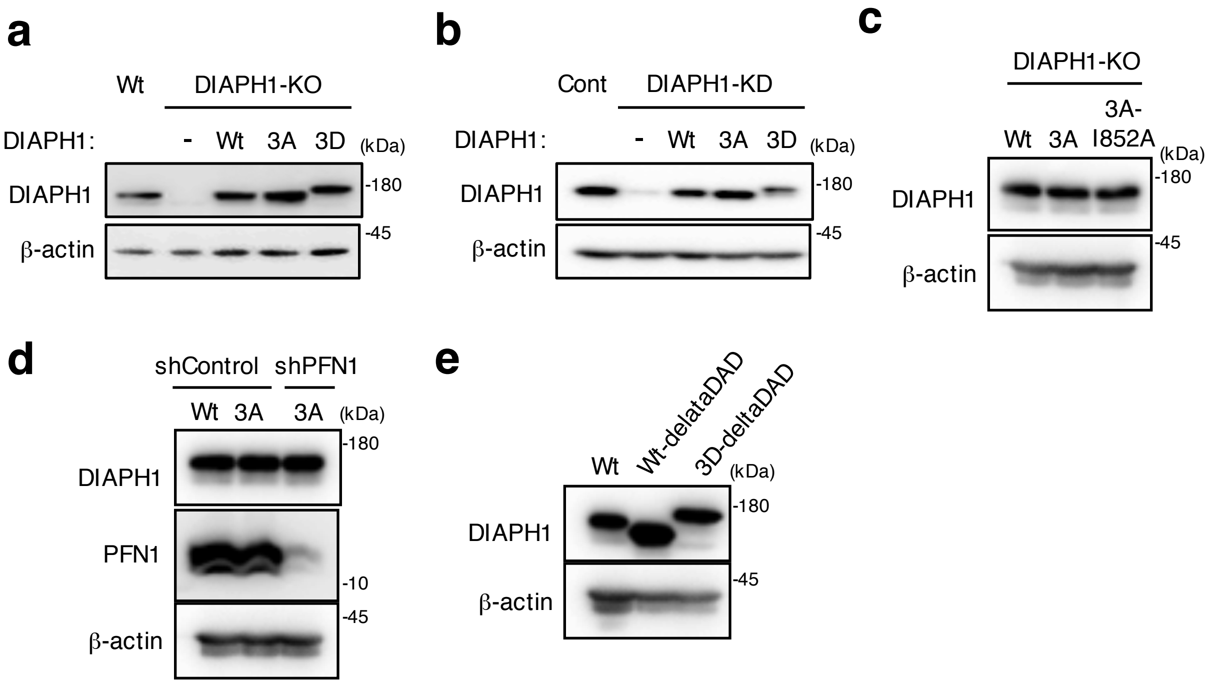

Supplementary Figure 4. Ectopic expression of wild-type DIAPH1 and its mutants in DIAPH1-KO HeLa cells

(a) Cell lysates were prepared from control (Wt) or H2B-mCherry-KO-Wt, -3A, or -3D cells and were subjected to immunoblotting using the indicated antibodies. **(b)** Inducible H2B-mCherry-KD-Wt, -3A, or -3D were treated with doxycycline (1μg/ml) for 24 hrs, and the cell lysates were subjected to immunoblotting using the indicated antibodies. **(c)** Whole cell lysates from Life-Act-mCherry-KO-Wt, -3A, or -3A/I852A mutants were subjected to immunoblotting using the indicated antibodies. **(d)** Whole cell lysates from Life-Act-mCherry-KO-Wt expressing tet-on shControl, -3A expressing tet-on shControl, or -3A expressing tet-on shPFN1 in the presence of 1 μg/ml doxycycline were subjected to immunoblotting using the indicated antibodies. **(e)** Whole cell lysates from Life-Act-mCherry-KO-Wt, -Wt/DAD, or -3D/DAD were subjected to immunoblotting using the indicated antibodies.

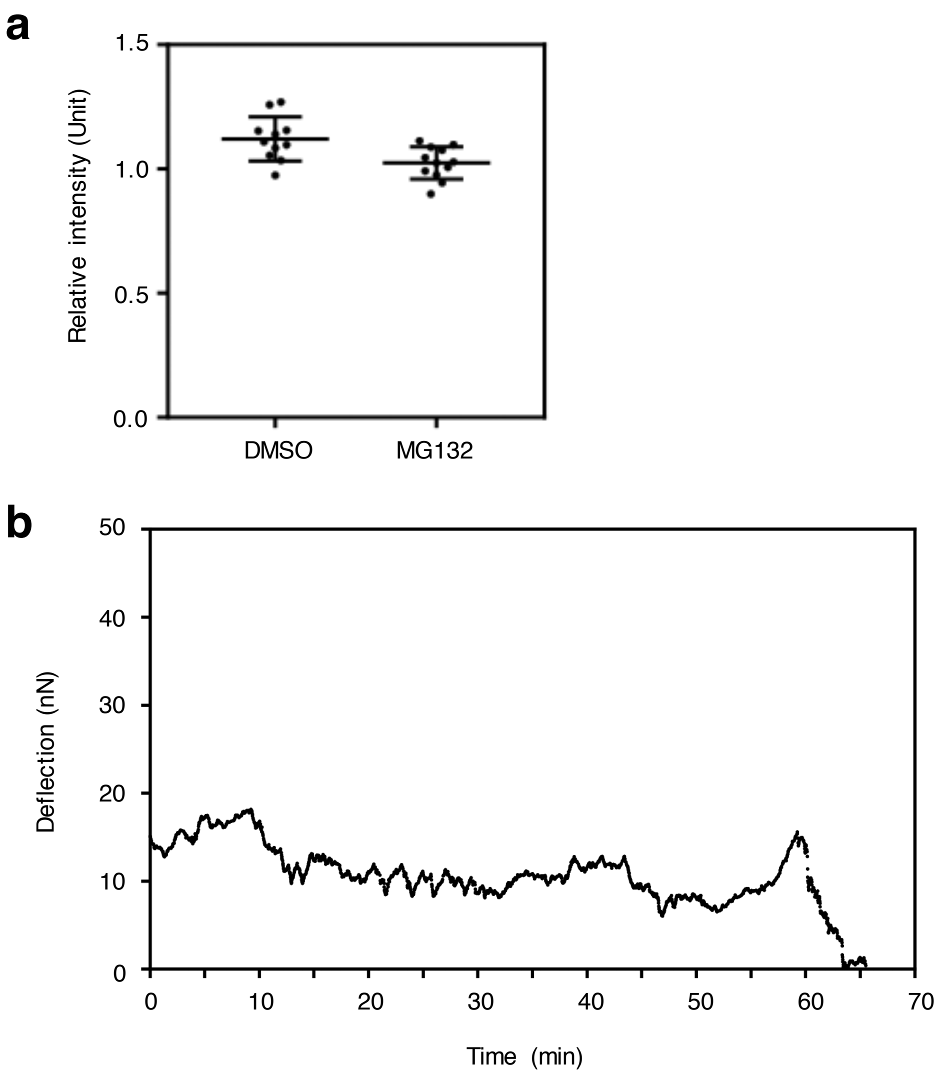

Supplementary Figure 5. Prolonged mitosis does not affect accumulation of F-actin on the cellular cortex and cortical tension

(a) Control HeLa cells expressing Life-Act-mCherry were stained with Hoechst 33342 for 10 min to monitor mitotic progression. Cells were then treated with either DMSO or MG132 (10 μM) and mitotic cells were imaged by microscopy without fixation. Relative signal intensities of mCherry at the cell cortex were quantified by Image J software. Black bars indicate means ±SD. DMSO: n=11, MG132: n=12. **(b)** Constant-height assay using AFM. The graph shows the upward deflection of the cantilever in the mitotic progression of control HeLa cells in the presence of MG132 (10 μM).

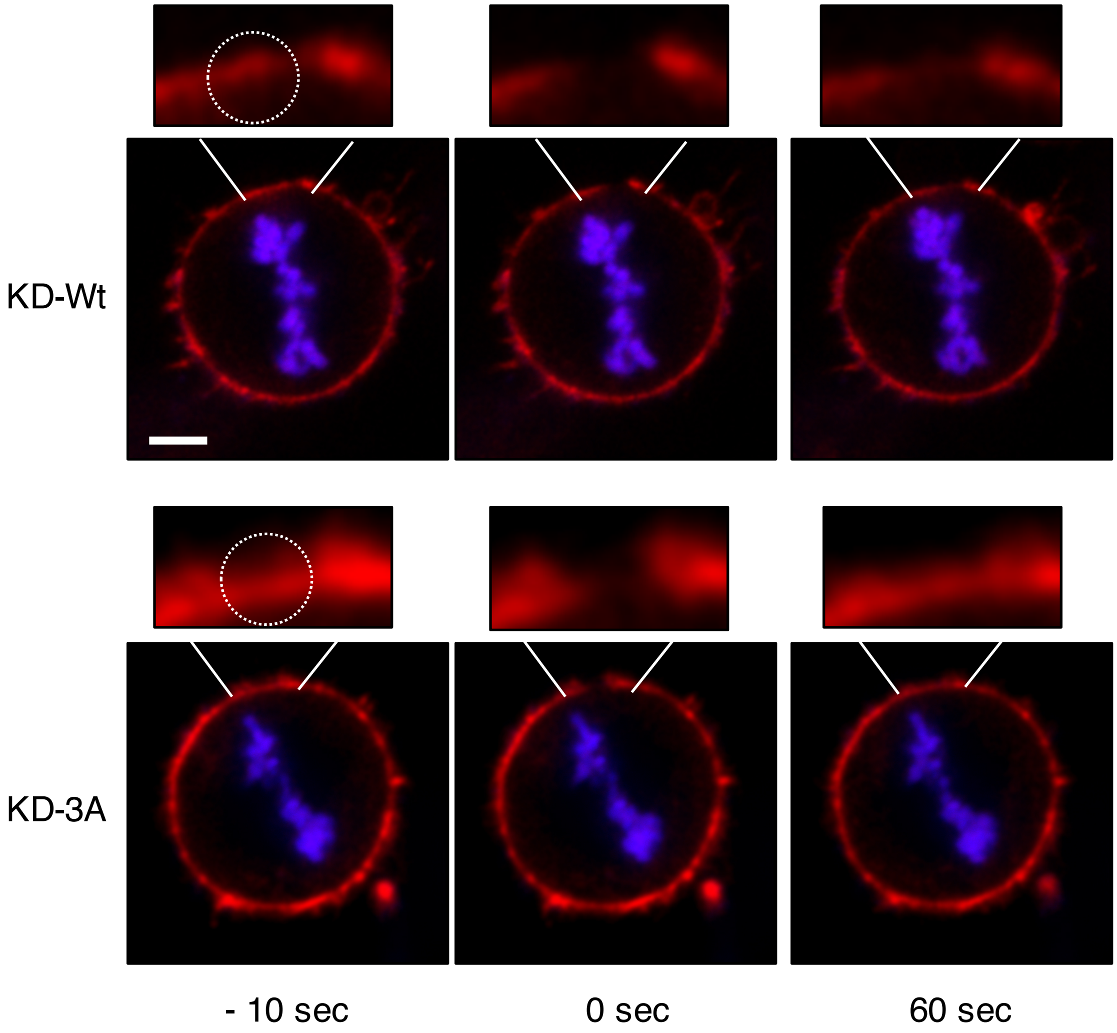

Supplementary Figure 6. Confocal microscope images of cells treated with SiR-Actin and Hoechst 33342 before and after laser irradiation

FRAP analysis was performed using LSM-780 inverted confocal microscope (Carl Zeiss) equipped with a GaAsp Detector. Cells were incubated with 100 nM SiR-Actin (Cytoskeleton Inc.) for 1 hr in order to visualize F-actin. Hoechst33342 was added to the medium 5 min before the imaging. A circular area of 1 μm diameter at the actin cortex of a metaphase cell was bleached with the 633 nm laser (100% laser power).

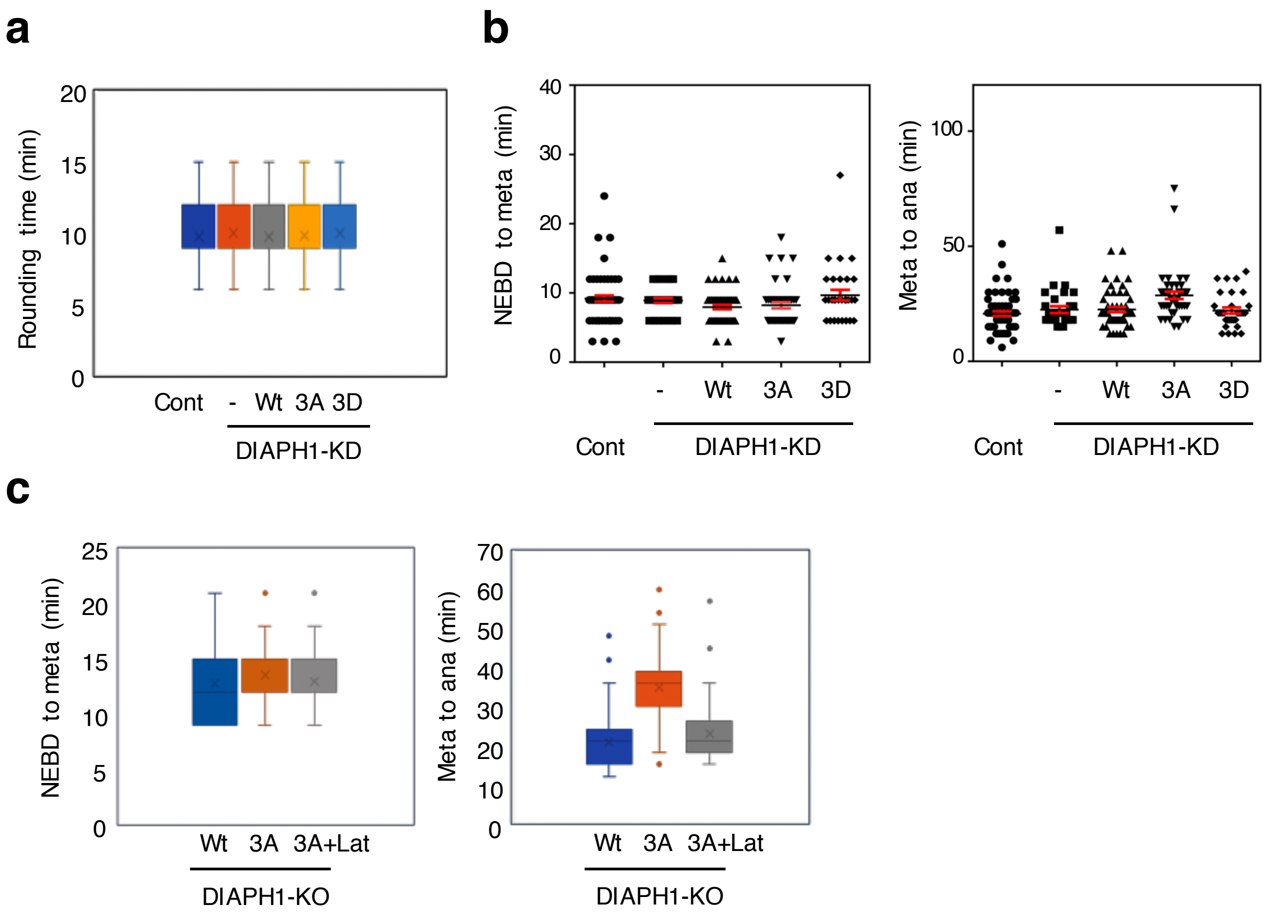

Supplementary Figure 7. Accumulation of F-actin and delayed onset of anaphase in KO-3A or KD-3A cells

(a) Control (Cont) or DIAPH1-KO HeLa cells expressing DIAPH1-Wt, -3A, or -3D mutants together with histone H2B-mCherry were imaged by time-lapse microscopy every 3 min (images were the same as that used for Figure 5a). The time interval between NEBD and mitotic rounding was determined. Cont: N=33, DIAPH1-KO: N=30, Wt: n=24, 3A: n=34, 3D: n=34. **(b)** Tet-on inducible Luciferase (Cont)- or DIAPH1 shRNA HeLa cells expressing mock (-), DIAPH1-Wt, -3A, or -3D together with Histone-H2B-mCherry were treated with doxycycline (1μg/ml) for 24 hrs, and then imaged by time-lapse microscopy every 3 min. The time duration between NEBD to metaphase or metaphase to anaphase was determined. Red bars with numbers indicate averages of the time length in minutes. Cont: n=59, DIAPH1-KD: n=31, Wt: n=53, 3A: n=45, 3D: n=31. **(c)** DIAPH1-KO cells expressing DIAPH1-Wt or -3A together with histone H2B-mCherry were treated with or without latrunculin A (1 μM) 30 min before analysis, and then imaged by time-lapse microscopy every 3 min. Wt: n=50, 3A: n=48, 3A (1 μM latrunculin A): n=52. Durations were determined as in **(a)** and colored bars indicate means ±SE.

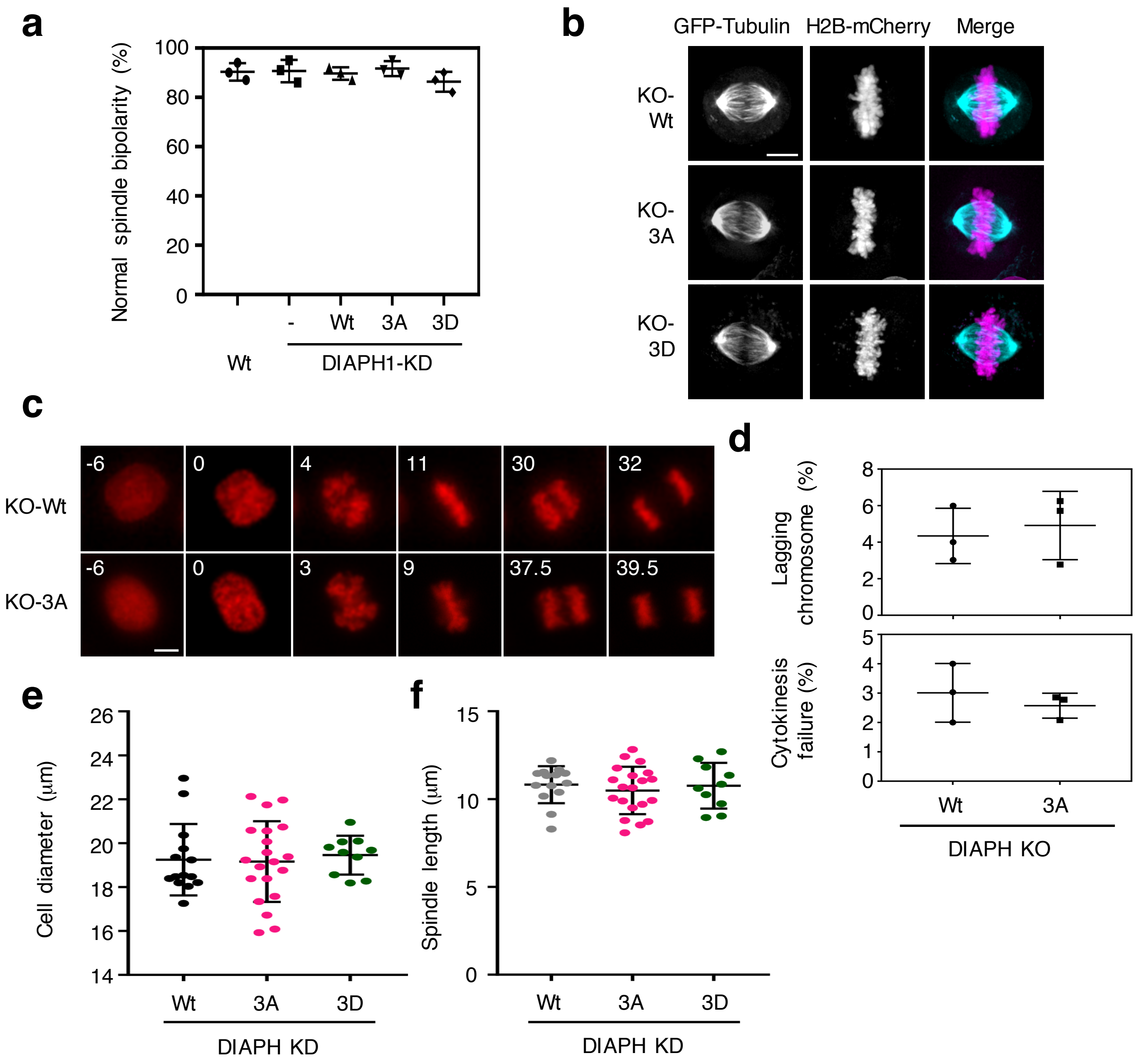

Supplementary Figure 8. DIAPH1-knockdown HeLa cells expressing DIAPH1-Wt, -3A, or -3D mutants show apparently normal spindle architecture, chromosome alignment, and cytokinesis

(a) Control (Wt) or KD-mock (-), -3A, or -3D expressing tubulin-EGFP were subjected to Hoechst 33342 staining for DNA, and observed by microscopy to quantify the number of cells (n=200) with normal spindle bipolarity in metaphase cells. The results were obtained from three independent experiments. Black bars indicate means ±SD. **(b)** Spindle architecture and chromosomes at anaphase of inducible H2B-mCherry-KD-Wt or -3A cells were analyzed. Scale bar, 5 mm. **(c)** Inducible H2B-mCherry-KD-Wt or -3A cells were subjected to live-cell imaging. Times after nuclear envelope breakdown are shown. Please see Supplementary Movies 1 and 2. Scale bar, 5 μm. **(d)** H2B-mCherry-KO-Wt or -3A cells were imaged by time-lapse microscopy every 3 min. The number of cells (n=200) with lagging chromosomes (upper) or cytokinesis failure (lower) were determined. Black bars indicate means ±SD. The results were obtained from three independent experiments. Cell diameter **(e),** Wt: n=14, 3A: n=20, 3D: n=10**,** or spindle length **(f),** Wt: n=24, 3A: n=20, 3D: n=10, of these cells were determined.

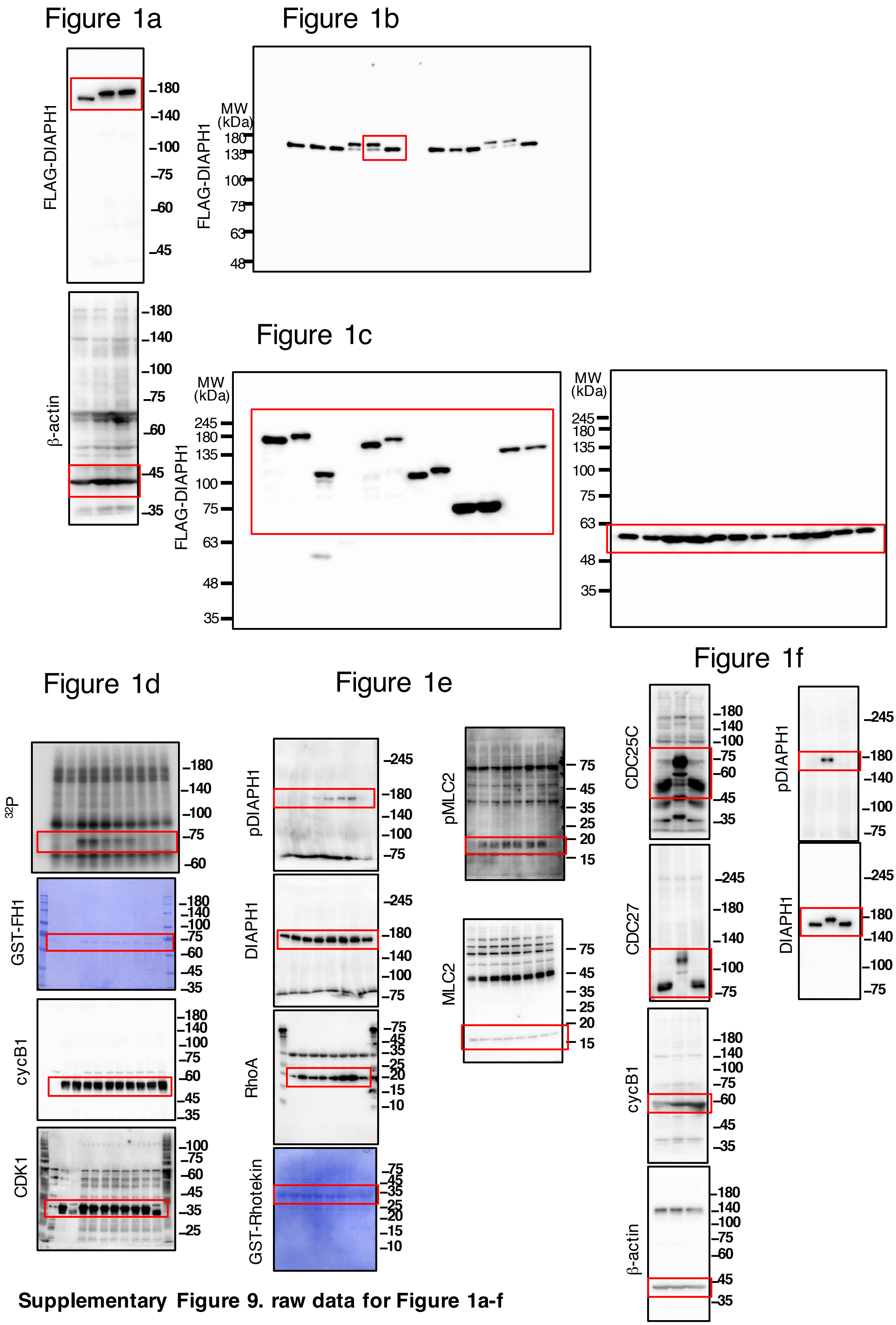

Supplementary Figure 9. raw data for Figure 1a-f

Figure 2a

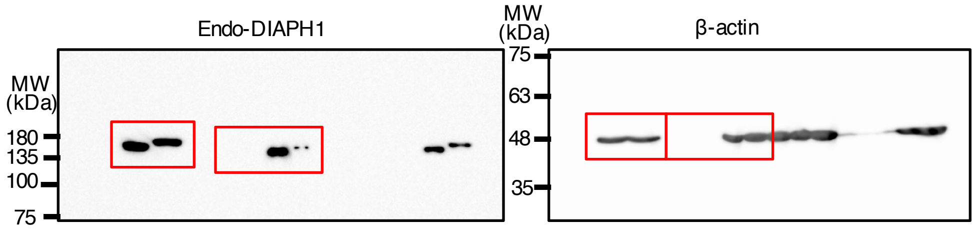

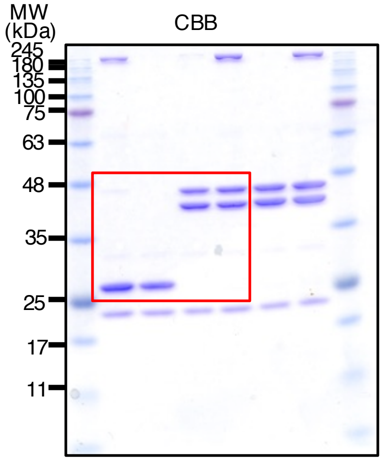

Figure 2b

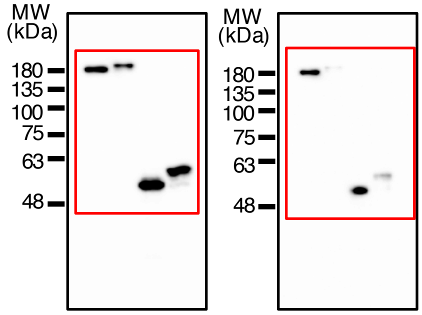

Figure 2c

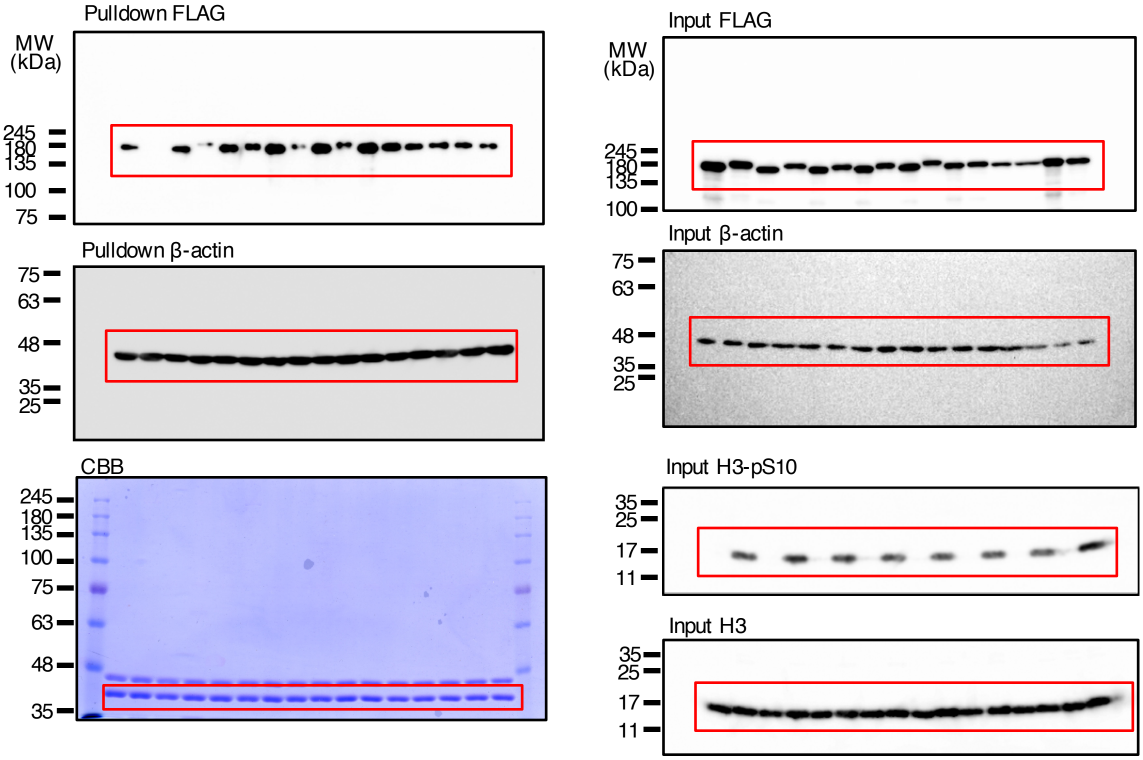

Supplementary Figure 9. raw data for Figures 2a-c

Figure 2d

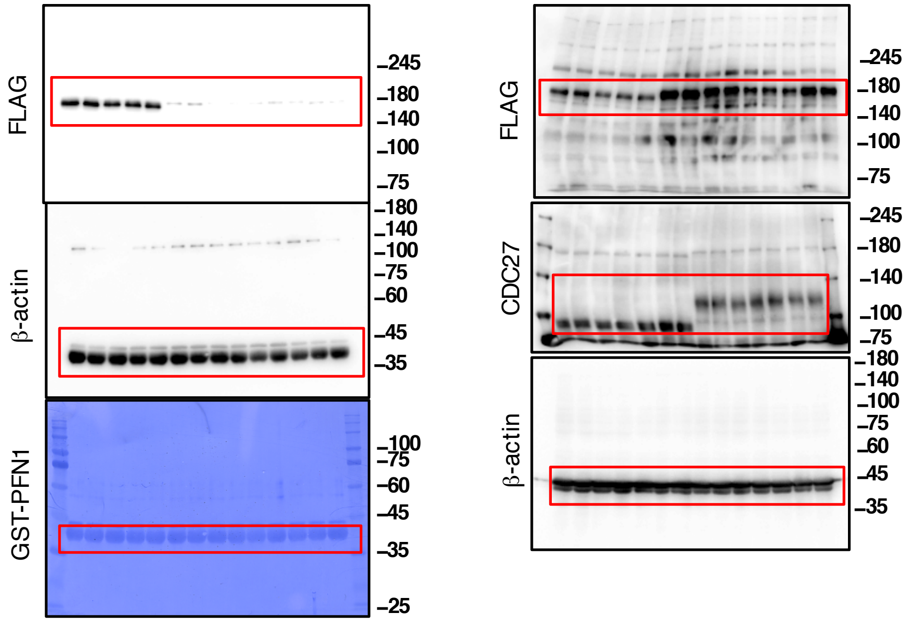

Supplementary Figure 9. raw data for Figures 2d

Supplementary Figure 1a

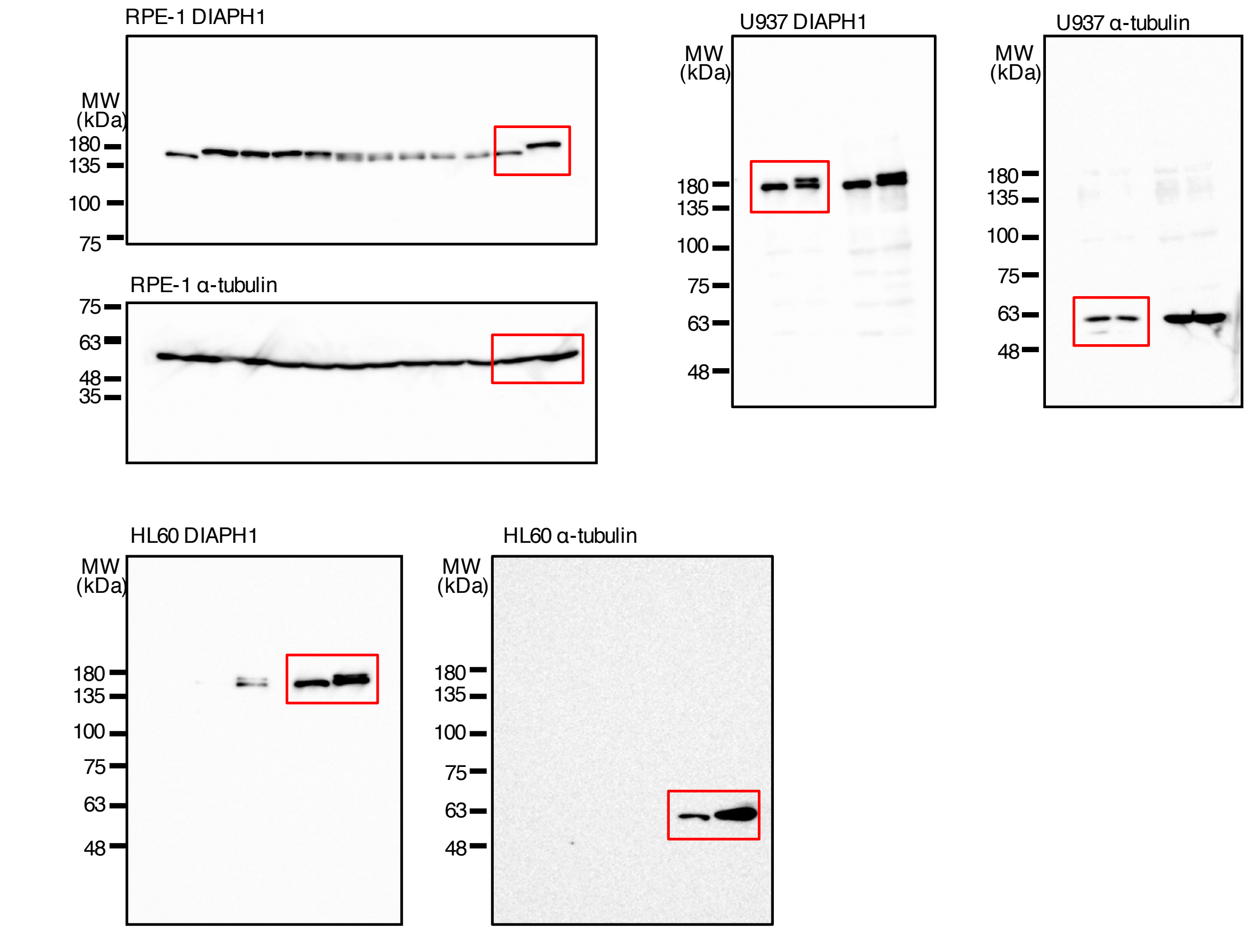

Supplementary Figure 1b

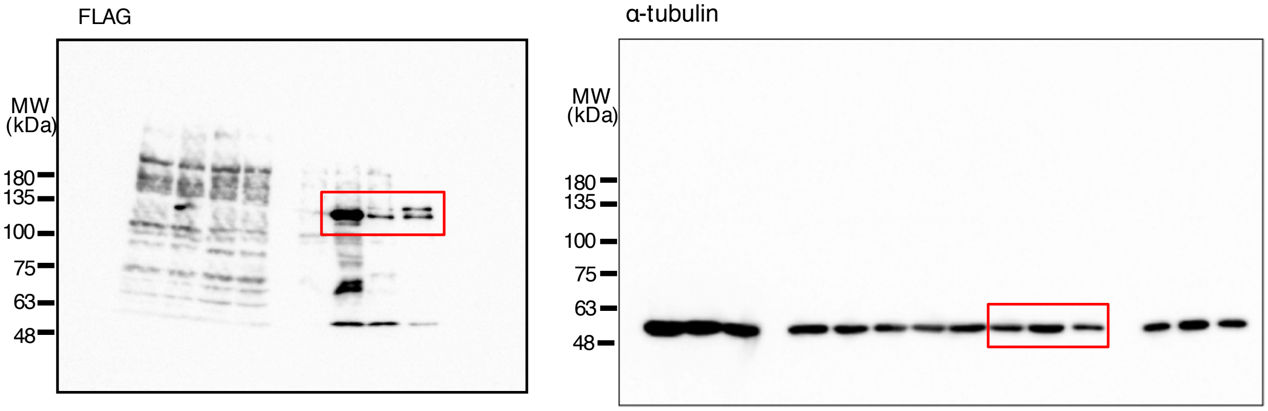

Supplementary Figure 9. raw data for Supplementary Figures 1a and 1b

Supplementary Figure 2b

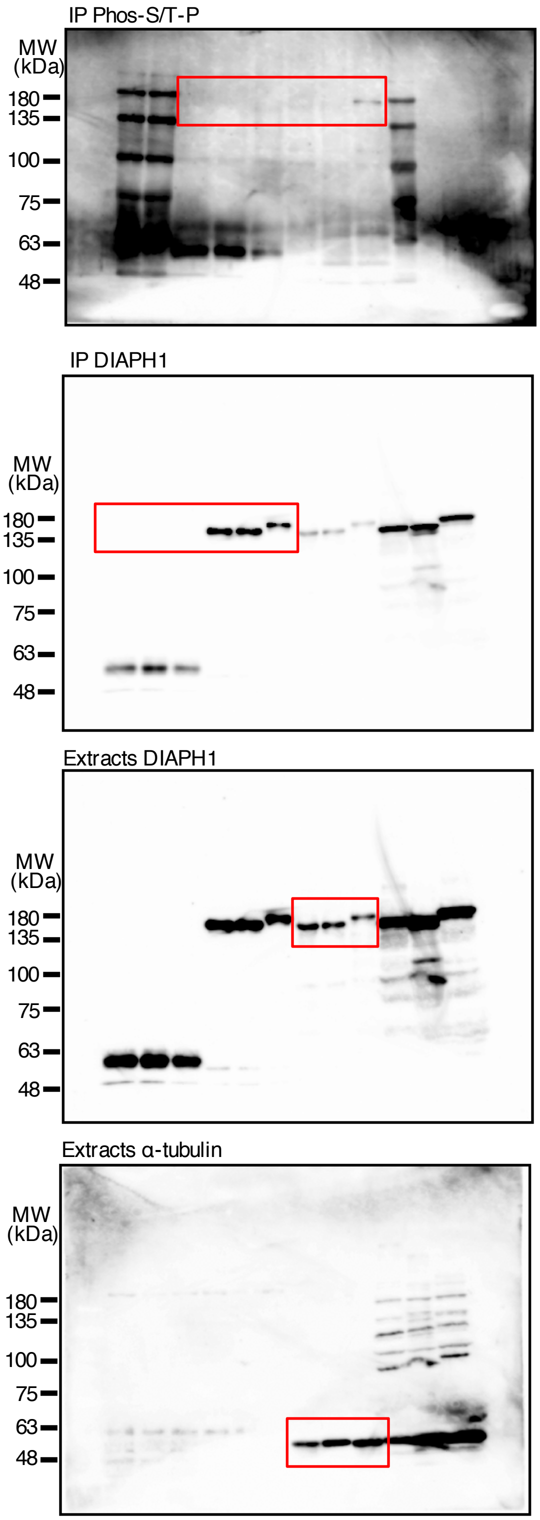

Supplementary Figure 9. raw data for Supplementary Figure 2b

Supplementary Figure 2c

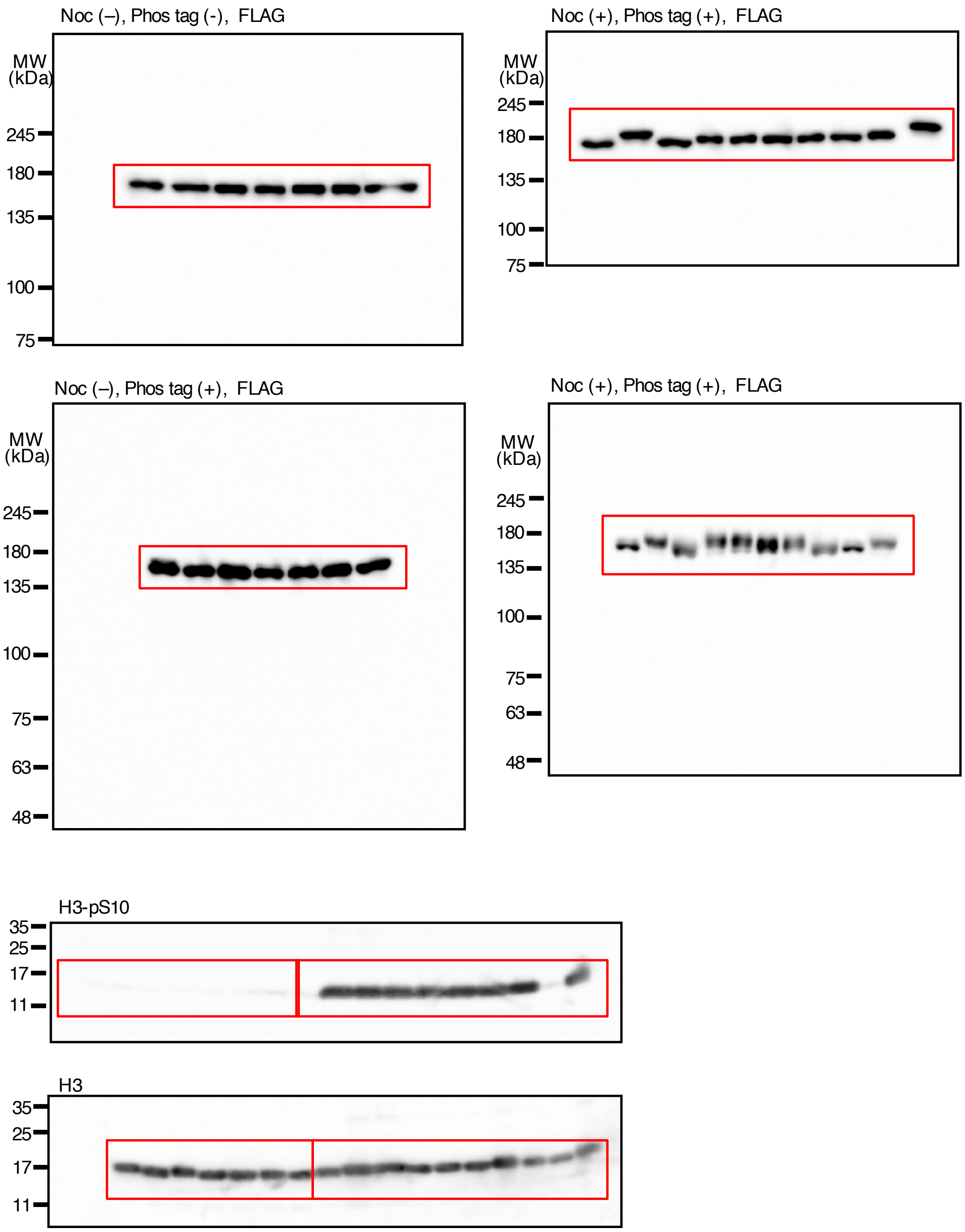

Supplementary Figure 9. raw data for Supplementary Figure 2c

Supplementary Figure 3b

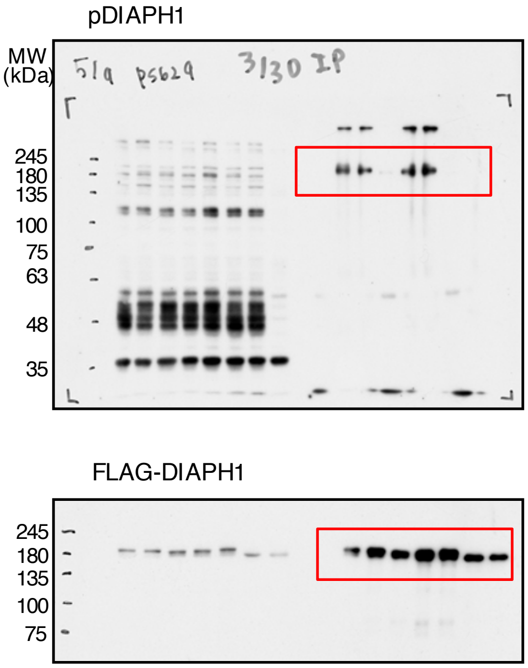

Supplementary Figure 3e

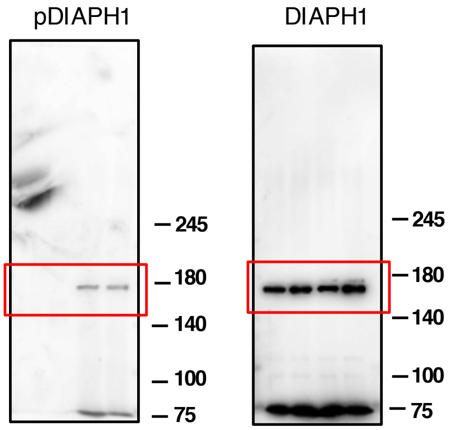

Supplementary Figure 9. raw data for Supplementary Figures 3b and 3e

Supplementary Figure 4a

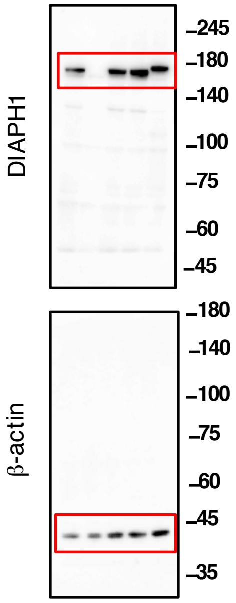

Supplementary Figure 4b

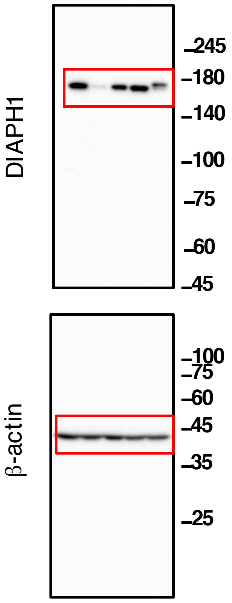

Supplementary Figure 4c

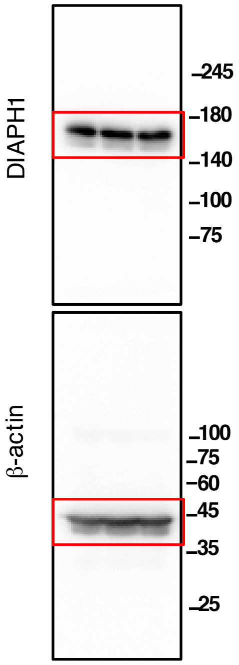

Supplementary Figure 4d

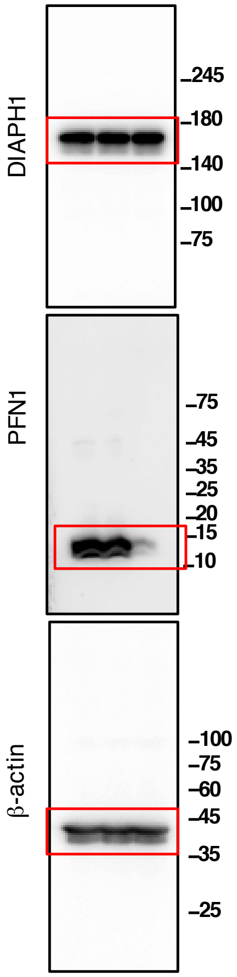

Supplementary Figure 4e

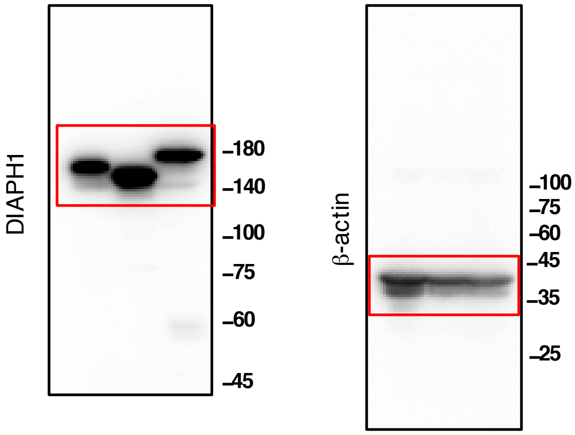

Supplementary Figure 9. raw data for Supplementary Figures 4a-e

Supplementary Table I A list of antibodies used in this study

Antibodies	Species	Source
Anti-Flag (M2)	Mouse	Sigma, St. Louis, MO
Anti-RhoA (67B9)	Rabbit	Cell Signaling Technology, Boston, MA
Anti-PFN1 (3237)	Rabbit	Cell Signaling Technology, Boston, MA
Anti-mDia1 (51)	Mouse	BD Transduction Laboratories, San Jose, CA
Anti-α-tubulin (T0926)	Mouse	Sigma, St. Louis, MO
Anti-β-Actin (AC-15)	Mouse	Abcam, Cambridge, United Kingdom
Anti-Phospho-S/T-P (MPM-2)	Mouse	Merck (Millipore), Darmstadt, Germany
Anti-Histone H3 (ab1791)	Rabbit	Abcam, Cambridge, United Kingdom
Anti-Histone H3-pSer10	Rabbit	Merck (Millipore), Darmstadt, Germany
Anti-DIAPH1-pSer629	Rabbit	This study
Anti-MAD2 (GTX104680)	Rabbit	GeneTex, Irvine, CA
Anti-BUBR1 (612502)	Mouse	BD Transduction Laboratories, San Jose, CA
Anti-CREST (15-234-0001)	Human	Antibodies Inc.
Anti-CDC25C (sc327)	Rabbit	Santa Cruz Biotechnologies, Santa Cruz, CA
Anti-CDC27 (AF3.1)	Mouse	Santa Cruz Biotechnologies, Santa Cruz, CA
Anti-CDK1 (H-297)	Rabbit	Santa Cruz Biotechnologies, Santa Cruz, CA
Anti-Cyclin B1 (H-433)	Rabbit	Santa Cruz Biotechnologies, Santa Cruz, CA
Anti-Hec1 (9G3.23)	Mouse	Novus Biologicals
Anti-Mlc2 (ab79935)	Rabbit	Abcam, Cambridge, United Kingdom
Anti-Phospho-Mlc2 (S19)(3671)	Rabbit	Cell Signaling Technology, Boston, MA
Anti-mouse IgG Alexa 568	Goat	Invitrogen
Anti-human IgG Alexa 488	Goat	Invitrogen
