# Supplementary material for: Cdk1-mediated DIAPH1 phosphorylation maintains metaphase cortical tension and inactivates the spindle assembly checkpoint at anaphase
Source: Nat Commun. 2019 Feb 28;10:981. doi: 10.1038/s41467-019-08957-w (PMC6395754; doi:10.1038/s41467-019-08957-w)
Supplement: Supplementary file 5 — Reporting Summary [file 41467_2019_8957_MOESM5_ESM.pdf]

nature research

Corresponding author(s): *Double-blind peer review submissions: write DBPR and your manuscript number here instead of author names.*

Last updated by author(s): *YYYY-MM-DD*

Reporting Summary

Nature Research wishes to improve the reproducibility of the work that we publish. This form provides structure for consistency and transparency in reporting. For further information on Nature Research policies, see Authors & Referees and the Editorial Policy Checklist.

Statistics

For all statistical analyses, confirm that the following items are present in the figure legend, table legend, main text, or Methods section.

n/a	Confirmed	
☐	☒	The exact sample size (n) for each experimental group/condition, given as a discrete number and unit of measurement
☐	☒	A statement on whether measurements were taken from distinct samples or whether the same sample was measured repeatedly
☐	☒	The statistical test(s) used AND whether they are one- or two-sided<br>*Only common tests should be described solely by name; describe more complex techniques in the Methods section.*
☒	☐	A description of all covariates tested
☒	☐	A description of any assumptions or corrections, such as tests of normality and adjustment for multiple comparisons
☐	☒	A full description of the statistical parameters including central tendency (e.g. means) or other basic estimates (e.g. regression coefficient) AND variation (e.g. standard deviation) or associated estimates of uncertainty (e.g. confidence intervals)
☒	☐	For null hypothesis testing, the test statistic (e.g. F, t, r) with confidence intervals, effect sizes, degrees of freedom and P value noted<br>*Give P values as exact values whenever suitable.*
☒	☐	For Bayesian analysis, information on the choice of priors and Markov chain Monte Carlo settings
☒	☐	For hierarchical and complex designs, identification of the appropriate level for tests and full reporting of outcomes
☒	☐	Estimates of effect sizes (e.g. Cohen's d, Pearson's r), indicating how they were calculated

Our web collection on statistics for biologists contains articles on many of the points above.

Software and code

Policy information about availability of computer code

Data collection	Zeiss LSM-780, -880, DeltaVision Elite, AxioVision, and BZ-9000 was used to acquire immunofluorescence images.<br>IX71 with MFP-3D was used for the force measurement.
Data analysis	ImageJ was used for image analysis.<br>GraphPad Prism 7 and 8, and Microsoft Excel were used for statistical analysis.<br>BZ-9000 analysis software was used for fluorescence image processing.<br>Imaris software version 7.6.4 were used for the measurement of the intra-kinetochore distances.

For manuscripts utilizing custom algorithms or software that are central to the research but not yet described in published literature, software must be made available to editors/reviewers. We strongly encourage code deposition in a community repository (e.g. GitHub). See the Nature Research guidelines for submitting code & software for further information.

Data

Policy information about availability of data

All manuscripts must include a data availability statement. This statement should provide the following information, where applicable:

- Accession codes, unique identifiers, or web links for publicly available datasets
- A list of figures that have associated raw data
- A description of any restrictions on data availability

Uncropped images of western blot data is available in supplementary figure 10-16.

Field-specific reporting

Please select the one below that is the best fit for your research. If you are not sure, read the appropriate sections before making your selection.

☒ Life sciences ☐ Behavioural & social sciences ☐ Ecological, evolutionary & environmental sciences

For a reference copy of the document with all sections, see nature.com/documents/nr-reporting-summary-flat.pdf

Life sciences study design

All studies must disclose on these points even when the disclosure is negative.

Sample size	Sample size was not predetermined by statistical methods as similar published researches.
Data exclusions	No data are excluded.
Replication	The number of samples and experiments were described in figure legend.
Randomization	Randomization was not applicable.
Blinding	Blinding was not applicable.

Reporting for specific materials, systems and methods

We require information from authors about some types of materials, experimental systems and methods used in many studies. Here, indicate whether each material, system or method listed is relevant to your study. If you are not sure if a list item applies to your research, read the appropriate section before selecting a response.

Materials & experimental systems

n/a	Involved in the study	
☐	☒	Antibodies
☐	☒	Eukaryotic cell lines
☒	☐	Palaeontology
☒	☐	Animals and other organisms
☒	☐	Human research participants
☒	☐	Clinical data

Methods

n/a	Involved in the study	
☒	☐	ChIP-seq
☒	☐	Flow cytometry
☒	☐	MRI-based neuroimaging

Antibodies

Antibodies used	Antibodies used in this study is summarized in Supplementary Table I.
Validation	All antibodies except for anti DIAPH1-pS629 are commercially available and validated by the companies (please see Supplementary Table I). An anti-DIAPH1-pS629 antibody was validated by a set of the experiments shown in the Supplementary Figures 3a-3e.

Eukaryotic cell lines

Policy information about cell lines

Cell line source(s)	HEK293T (293T; ATCC) , HeLa (ATCC) cells, Lifeact-mCherry-HeLa Kyoto cells (provided by Dr. Cedric Cattin and Dr. Daniel J. Muller), Histone H2B-mCherry/Tubulin-LAP-HeLa Kyoto cells (also provided by Dr. Cedric Cattin and Dr. Daniel J. Muller)
Authentication	All cell lines were not authenticated.
Mycoplasma contamination	All cell lines were not tested for mycoplasma contamination.
Commonly misidentified lines (See ICLAC register)	*Name any commonly misidentified cell lines used in the study and provide a rationale for their use.*
